# Biological treatments for zoonotic salmonellosis: an evolving therapeutic landscape

**DOI:** 10.3389/fmicb.2026.1769519

**Published:** 2026-02-17

**Authors:** Sushan Ma, Xingmei Liu, Min Shen, Shanshan Deng, Chengxiang Ding, Xin Yang, Lin Zhang, Xu Jia

**Affiliations:** 1 Key Laboratory of Non-coding RNA and Drug Discovery at Chengdu Medical College of Sichuan Province, School of Basic Medical Sciences, Chengdu Medical College, Chengdu, Sichuan, China; 2 Vincent Mary School of Engineering, Science and Technology, Assumption University of Thailand, Bangkok, Thailand; 3 Academy of Animal Husbandry and Veterinary Science, Qinghai University, Xining, Qinghai, China; 4 Department of Pharmacy, ShaoXing People’s Hospital, ShaoXing Hospital, ZheJiang University School of Medicine, Shaoxing, Zhejiang, China

**Keywords:** bacteriophages, biotherapies, probiotics, *Salmonella*, salmonellosis, vaccines

## Abstract

Salmonellosis, a predominant food-borne gastroenteric disease, presents a substantial and escalating threat to global public health, largely attributable to infections by non-typhoidal *Salmonella* serovars such as *Salmonella enterica* serovar *Typhimurium* (*S. Typhimurium*) and *Salmonella* serovar *Enteritidis* (*S. Enteritidis*). The conventional reliance on antimicrobial agents for treating salmonellosis is increasingly compromised by the emergence and spread of multidrug-resistant (MDR) strains, necessitating an urgent shift toward alternative therapeutic strategies. In recent years, biological therapies, including bacteriophages, probiotics, vaccines and their synergistic combinations, have demonstrated considerable promise. Advances in antibacterial research highlight the potential of biotherapies to offer high efficacy with minimal side effects. This review consolidates the most current information on the methodologies, mechanisms of action, functional benefits, and clinical research progress of these biological treatments in combating zoonotic salmonellosis. We delve into recent innovations such as engineered phages and probiotics, postbiotics, novel vaccine platforms including mRNA and nanoparticle-based delivery systems, and the development of multivalent vaccines. Furthermore, the importance of the One Health perspective in controlling salmonellosis and the translational challenges, including regulatory and commercialization hurdles, are discussed. It is anticipated that biotherapies, particularly engineered and combination approaches, hold significant potential for addressing the challenge of MDR bacteria and safeguarding public and animal health.

## 1 Introduction

### 1.1 *Salmonella* and salmonellosis: an enduring global health challenge

*Salmonella* is a pathogenic bacterium isolated from pig intestines during the cholera epidemic of 1885 (Steele, 1963). *Salmonella* is a gram-negative bacterium that belongs to the family Enterobacteriaceae and is an acidophilic facultative anaerobic bacterium that can rely on systemic flagellar motility (GBD 2017 Non-Typhoidal Salmonella Invasive Disease Collaborators, 2019). Infection with *Salmonella* typically presents with nonspecific clinical symptoms (Popa and Papa, 2021). To date, more than 2,600 serotypes of *Salmonella* have been detected (Teklemariam et al., 2023). *S. bongori* was the first subspecies isolated, and *S. Enteritidis* and *S. Typhimurium* were the two most common serotypes, followed by *S*. *Newport*, *S*. *Javiana*, and *S*. *Heidelberg* (Dos Santos et al., 2019; Oh and Park, 2017). The global burden of invasive non-typhoidal *Salmonella* (iNTS) disease is considerable, contributing to significant morbidity and mortality, particularly among vulnerable populations. By contrast, non-typhoidal *Salmonella* (NTS) more commonly causes gastroenteritis. In the United States alone, *Salmonella* is estimated to cause about 1.35 million infections, 26,500 hospitalizations, and 420 deaths annually (Acevedo-Villanueva et al., 2021).

Different serotypes of *Salmonella* lead to differential clinical manifestations. *S. Enteritidis* infection typically causes diarrhea, abdominal pain, nausea, vomiting, and *fever*. *S. Typhimurium* causes mainly acute infectious diseases, such as gastroenteritis and septicaemia. The symptoms of the gastroenteritis type are similar to those of *S. Enteritidis*, and the septicemia type may cause symptoms of systemic infection (Ferrari et al., 2019). *Salmonella* is widely distributed in nature, spreads easily, has many types, and is difficult to eradicate (Thomas et al., 2013). This diversity implies that single, serotype-specific control measures may have limited overall impact, necessitating broader or combined therapeutic and preventive approaches. Its zoonotic characteristic, with transmission occurring between animals and humans, underscores the need for control efforts that span human, animal, and environmental domains, aligning with the one health concept.

Salmonellosis imposes a significant economic burden, with estimated annual costs in the United States rising from $1 billion in 1987 to approximately $3.3 billion in recent years, ranking it third among foodborne pathogens (Lobertti et al., 2024). These estimates likely underrepresent the true burden, as they often exclude intangible costs such as pain and suffering, lost leisure time and long-term health consequences. Additionally, losses in the agricultural sector due to reduced animal productivity, regulatory interventions and product recalls further underscore the need for cost-effective and sustainable control strategies. This significant economic toll highlights the urgent need for cost-effective and sustainable control measures. Biological therapies, if proven to be more effective or sustainable than current methods, particularly in the face of antimicrobial resistance, could offer substantial economic benefits by reducing healthcare expenditures and productivity losses, thereby justifying continued investment in their research and development.

### 1.2 The rise of antimicrobial resistance and tolerance: the imperative for alternative therapies

Since their discovery in the twentieth century, antibiotics have been the mainstay of treatment for salmonellosis. For example, Conventional antibiotics including chloramphenicol (inhibiting peptide chain elongation to block protein synthesis) (Kunstadter et al., 1951); quinolone antibiotics (levofloxacin, ciprofloxacin) interfere with bacterial growth and reproduction by preventing DNA replication and RNA transcription (Macías-Farrera et al., 2018); third-generation cephalosporins inhibit cell wall synthesis and thus exert an inhibitory effect (Schmidt et al., 2022); and different antibiotics have shown good therapeutic effects on *Salmonella* (Braetz et al., 2022; Gebeyehu et al., 2022; Jacks et al., 1981). However, the widespread and often injudicious use of antibiotics in human and veterinary medicine has led to a dramatic increase in antimicrobial resistance (AMR) (Feye et al., 2016). *Salmonella* strains undergo genetic mutations, rendering them resistant to antibiotics to which they were previously susceptible, leading to bacterial populations with increased overall resistance (Mathew et al., 2009). Aslam et al. reported a high prevalence of antimicrobial resistance among *Salmonella* isolates recovered from retail meats, with multidrug resistance being frequently observed. Resistance was particularly common to several clinically and veterinary important antimicrobials, and distinct resistance gene profiles were associated with specific *Salmonella* lineages, indicating that foodborne *Salmonella* constitutes a significant reservoir of antimicrobial resistance (Aslam et al., 2012).

Beyond antimicrobial resistance, antibiotic tolerance has increasingly been recognized as a distinct and clinically relevant contributor to treatment failure in *Salmonella* infections (Fanous et al., 2025). Tolerance is mainly manifested as the prolonged survival time of bacteria under antibiotic exposure (Pontes and Groisman, 2019). This physiological state markedly diminishes the efficacy of many conventional antibiotics whose bactericidal activity depends on active cellular processes, including cell wall synthesis, DNA replication, and protein translation. As a consequence, intracellular *Salmonella* can survive prolonged antibiotic treatment, contributing to delayed clearance, relapse, and chronic infection. Recent studies have highlighted antibiotic tolerance as a major physiological consequence of *Salmonella*’s adaptation to the intracellular niche and a key determinant of treatment failure despite apparent *in vitro* susceptibility (Wijburg et al., 2000; Fields et al., 1986). This phenomenon poses a serious threat to global public health and could cause approximately 10 million deaths annually by 2050 if it is not effectively controlled (Munshi et al., 2025; Lamichhane et al., 2024).

### 1.3 Biological therapies: a promising frontier

In response to the AMR crisis, biological therapies, including bacteriophages (phages) (Kortright et al., 2019), probiotics (Aghamohammad and Rohani, 2023), and vaccines (Frost et al., 2023), have emerged as promising alternatives for the treatment and control of salmonellosis. These approaches offer the potential for high efficacy with minimal side effects compared to conventional antibiotics. Generally, phages function by directly destroying the bacterial cell structure; probiotics modulate the intestinal environment, compete with pathogens, and stimulate host immunity; and vaccines induce specific, long-lasting immune protection. The “promise” of these biotherapies extends beyond their direct antimicrobial effects. Their potential for more targeted action. This review aims to compile the most up-to-date information on these biotherapeutic modalities for zoonotic salmonellosis, encompassing their methods of application, underlying mechanisms of action, functional benefits, and progress in clinical research. Furthermore, it seeks to provide a theoretical foundation for future research directions and facilitate the establishment of safer, more effective *Salmonella* control systems.

## 2 Bacteriophage therapy for salmonellosis

### 2.1 Fundamentals of phage biology and lytic action

**Figure 1:**
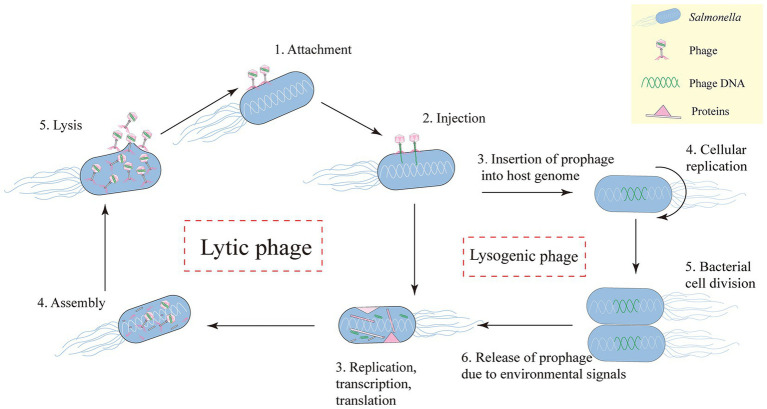
*Salmonella* phage infection follows five key steps: (1) Targeted attachment to bacterial receptors (LPS/outer membrane proteins) via tail proteins; (2) Genome injection through membrane penetration; (3) Host takeover for viral replication and lytic enzyme production; (4) Progeny assembly via genome packaging and capsid formation; (5) Cell lysis through endolysin/holin action, releasing 50–200 new phages. Lytic phages complete this cycle in 20–40 min, while lysogenic types integrate as prophages until activated by stress.

Bacteriophages are the most ubiquitous biological organisms in nature. They are viruses that can infect bacteria (Dion et al., 2020). Compared with broad-spectrum antibiotics, phages have high specificity and do not damage coexisting bacterial flora; they have the advantages of wide distribution, simple isolation, a short development cycle, low cost, relatively strong environmental stability under specific conditions, and low toxicity and side effects (Doss et al., 2017; Strathdee et al., 2023). Bacteriophages are divided into lytic phages (which attach to host cells and replicate by releasing new phages) and temperate phages (which integrate their genome into the host cell and replicate) (Hobbs and Abedon, 2016) (Figure 1).

For therapeutic applications, strictly lytic phages are overwhelmingly preferred due to significant safety concerns associated with temperate phages. Temperate phages carry the inherent risk of horizontal gene transfer, potentially transferring virulence factors or antibiotic resistance genes from the phage genome or the host bacterium to other bacteria (Monteiro et al., 2019). Furthermore, their lytic conversion is unpredictable and dependent on environmental cues, which compromises treatment reliability. Consequently, lytic phages, which offer immediate and controlled bactericidal effects without the risk of genetic transfer, are the exclusive choice for current clinical applications, unless lysogenic variants are genetically modified to irreversibly disable their integration capabilities. This fundamental safety consideration dictates phage selection criteria and informs strategies for phage engineering (Dion et al., 2020).

### 2.2 Applications of lytic phages

#### 2.2.1 Single phage interventions

Extensive research has demonstrated the efficacy of phage therapy through its lytic action against pathogenic bacteria, effectively suppressing their growth and proliferation (Campbell and Rolfe, 1975). The bactericidal strategies employed by phages are diverse, as summarized in Table 1. Robert et al. reported a significant decrease in light intensity within 1 min after spraying the lytic phages Eϕ151 or Tϕ7 onto the skin of chickens infected with bioluminescent *S. Enteritidis* P125109 or *S. Typhimurium* 4/74, respectively (Atterbury et al., 2020), demonstrating the lytic activity of the phages against *Salmonella*. Similar effective lysis by the phage was observed in milk or eggs contaminated with *Salmonella* (Modi et al., 2001; García et al., 2015; Li et al., 2020). Phages can decrease the *in vivo* colonization of *Salmonella*. Mengzhe et al. reported that administering phage STP4-a to *S. Typhimurium* ATCC14028-infected chickens for 7 consecutive days resulted in a reduction in bacterial counts in the feces, which could be attributed to the ability of the phage to catch and kill bacteria in the intestine, subsequently preventing bacterial colonization (Li et al., 2020). MiJin et al. reported a decrease in the expression of pro-inflammatory cytokines such as IL-6, IFN-*γ* and TNF-*α* and an increase in the expression of anti-inflammatory cytokines such as IL-4 and HSP27 after *Salmonella*-infected laying hens were treated with the BF2165 phage. This outcome was attributed to the ability of the phage to inhibit the colonization of *Salmonella*, thereby reducing the inflammatory burden on tissues caused by *Salmonella* (Lee et al., 2021). These findings demonstrate that phage therapy not only directly eliminates *Salmonella* but also modulates host immune responses by rebalancing pro-and anti-inflammatory cytokine networks.

**Table 1:** Summary of lytic phage therapies for salmonellosis.

*Salmonella* serotypes	Phage	Function	References
Single phage			
*S. Enteritidis*	Phage SJ2	The lytic phage SJ2 lysed *S. Enteritidis* in milk and cheese, reducing the survival ability of *S. Enteritidis*.	Modi et al. (2001)
*S. Enteritidis* P125109	Phage Eϕ151	The lytic phage Eϕ151 and Tϕ7 lysed the *S. Enteritidis* P125109 and *S. Typhimurium* 4/74 on the surface of chicken skin, respectively.	Atterbury et al. (2020)
*S. Typhimurium* 4/74	PhagesTϕ7		
Multiple serotypes of *Salmonella*	Phage STP4-a	In an in vitro model, phage STP4-a has a significant antibacterial activity on different serotypes of *Salmonella*, such as *S. Typhimurium*, *S. Enteritidis*, S. Newport, S. Heidelberg and S. Poona. In an in vivo model, phage STP4-a administered orally rapidly captured and eradicated multiple *Salmonella*, including *S. Typhimurium* CMCC 50115, *S. Enteritidis* CMCC 50041, *S. Paratyphi* ACMCC 50001, *S. Paratyphi* BCMCC 50094 and *S. Typhimurium* CMCC 50071, thereby preventing their colonization in the gastrointestinal tract.	Li et al. (2020)
*S. Enteritidis* CVCC1806	Phage PSE-D1	Two broad-spectrum phages, PSE-D1 and PST-H1, exhibited significant inhibitory effects on the growth of *S. Enteritidis* CVCC1806 and *S. Typhimurium* CVCC3384, respectively. Furthermore, PSE-D1 and PST-H1 demonstrated pronounced lytic activity against *S. Enteritidis* CVCC1806 and *S. Typhimurium* CVCC3384, respectively.	Cao et al. (2022)
*S. Typhimurium* CVCC3384	Phage PST-H1		
*S. Enteritidis* DR016	Phage vB_Sen_STGO-35-1	Phage vB_Sen_STGO-35-1 can lyse *S. Enteritidis* DR016, suggesting a potential counter resistance mechanism that enables phage vB_Sen_STGO-35-1 to overcome the resistance developed by *S. Enteritidis* DR016 effectively.	Barron-Montenegro et al. (2022)
S. Thompson T10 S. Mbandaka	Phage MSP1	The broad-spectrum phage MSP1 was demonstrated efficacy in reducing the viable counts of S. Thompson T10 and S. Mbandaka in both chicken and milk, which also exhibiting significant inhibition and clearance capabilities against biofilms.	Park et al. (2023)
*S. Typhimurium* ATCC14028	Phage T102	Phage T102 effectively inhibited and cleared biofilms produced by multidrug-resistant *S. Typhimurium* ATCC14028.	Ding et al. (2023)
S. Enteriditis EG.SmE1 *S. typhimurium* EG.SmT3	Phage LPSent1	Phage LPSent1 lysed both *S. Enteritidis* EG.SmE1 and *S. Typhimurium* EG.SmT3, and effectively inhibited and eliminated the biofilms produced by these strains.	Al-Hindi et al. (2023)
*S. typhimurium* Kol-551	Phage STWB21	Phage STWB21 reduced the number of S. Typhimurium Kol-551 colonies in the liver and spleen of mice.	Mondal et al. (2023)
*S. Typhimurium* ATCC14028	Phage BF2165	Supplementation of chicken feed with phage BF2165 effectively inhibited the colonization of *S. Typhimurium* ATCC14028 in the spleen, oviduct, cecum and feces. Furthermore, phage BF2165 alleviated the host inflammatory response induced by *S. Typhimurium* ATCC14028 through reducing the expression of IL-6 and IFN-γ in the jejunum, and IFN-γ and TNF-α in the liver of laying hens.	Lee et al. (2021)
Microencapsulated phage			
*S. Typhimurium* ATCC13311	Phage T156	Compared to the free phage T156, microencapsulated phage T156 with enhanced adsorption efficiency and lysis rate exhibits pronounced efficacy in lysing *S. Typhimurium* ATCC13311 present in milk.	Li et al. (2021)
*S. Typhimurium* FSL S5-370 *S. Enteritidis* FSL S5-371	Phage SPT 015	Microencapsulated phage SPT015 displayed increased stability and effectively reduced the viable bacterial counts of both *S. Typhimurium* FSL S5-370 and *S. Enteritidis* FSL S5-371.	Petsong et al. (2021)
*S. Enteritidis* CICC10467	Phage XG-SA-CaCl_2_-COS	In comparison to the free phage XG-SA-CaCl_2_-COS, the microencapsulated phage XG-SA-CaCl_2_-COS exhibits superior inhibitory effects against *S. Enteritidis* CICC10467, along with enhanced stability and convenience in storage.	Zhang et al. (2022)
S. Senftenberg	Phage FGS011	Oral administration of microencapsulated phage FGS011 to chickens infected with S. Senftenberg facilitates targeted delivery of the phage to the cecum, the primary colonization site, where it exerts lytic activity and enhances the efficacy of phage therapy.	Lorenzo-Rebenaque et al. (2022), Lorenzo-Rebenaque et al. (2021)
Phage cocktail			
*S. Enteritidis* SE006	Phage GSP162 Phage GSP193 Phage GSP001 Phage GSP032	The in vivo and in vitro experiments demonstrated the enhanced efficacy of the phage cocktail composed of phages GSP162, GSP193, GSP001 and GSP032 against *S. Enteritidis* SE006, thereby significantly delaying the emergence of phage-resistant *Salmonella* mutants.	Gao et al. (2022)
*S. Typhimurium* S. Coleraesius *S. Infantis* S. Cerro *S. Typhimurium* ILSI	Phage cocktail ECPS-6	The phage cocktail ECPS-6 exhibits greater lytic activity against various *Salmonella* serotypes compared to individual phage.	Aguilera et al. (2022)
*S. Enteritidis*	Phage cocktail SalmoFREE^®^	Adding the phage cocktail SalmoFREE^®^ to chicken feed effectively reduced the number of viable *S. Enteritidis* and inhibited the development of antibiotic resistance in *S. Enteritidis*.	Clavijo et al. (2019)
S. Newport *S. Typhimurium* S. Thompson S. Heidelberg *S. Enteritidis* *S. Typhimurium*	Salmonelex™ (a commercially available *Salmonella* specific phage cocktail)	The phage cocktail Salmonelex can decrease the number of *Salmonella* in ground chicken production.	Grant et al. (2017)
*S. Typhimurium* SL1344 *S. Typhimurium* S01160-12	Phage SPFM2 Phage SPFM10 Phage SPFM14	The in vivo assay showed that the combination of phages SPFM10 and SPFM14 as well as SPFM2 exhibited enhanced antimicrobial activity, which can effectively reduced the colonization of both *S. Typhimurium* SL1344 and *S. Typhimurium* S01160-12 in larvae.	Thanki et al. (2022)
*S. Typhimurium* *S. Enteritidis*	Phage vB_SenM2 and vB_Sen-TO17	The phage cocktail vB-SenM2 and vB-Sen-TO17 was effective in clearing biofilm and in reducing the bacterial load of *S. Typhimurium* and *S. Enteritidis* in chicken gut.	Kosznik-Kwaśnicka et al. (2022)
Phage-antibiotic			
*S. Paratyphi*ANA3	Phage KM16 (Kanamycin)	The combination of phage KM16 with kanamycin sulfate against *S. Paratyphi* A NA3 for 12 h exhibited better antimicrobial efficacy compared to the kanamycin alone.	Jiang et al. (2021)
*S. Typhi* *S. Typhimurium* *S. Enteritidis* *S. Gallinarum* S. Blegdam I S. Blegdam II	Phage ZCSE9 (Kanamycin)	The combination of phage ZCSE9 and kanamycin showed a higher antibacterial effect against multiple *Salmonella* serotypes including *S. Typhi*, *S. Typhimurium*, *S. Enteritidis*, *S. Gallinarum*, S. Blegdam I and S. Blegdam II than either phage ZCSE9 or kanamycin alone.	Abdelsattar et al. (2023)
*S. Enteritidis* PT1	Phage phiPT1 (cefixime, gentamicin, ciprofloxacin, aztreonam)	In contrast to the utilization of phage phiPT1 alone, phage phiPT1 can exert enhanced antibacterial activity and improved therapeutic effect on *S. Enteritidis* PT1 when combined with cefixime, gentamicin, ciprofloxacin and aztreonam, respectively.	Alharbi et al. (2023)
*S. Typhimurium* CGMCC 1.1174	Phage ST-3 (levofloxacin, ciprofloxacin)	The combination of phage ST-3 with levofloxacin enhanced the clearance to *S. Typhimurium* CGMCC 1.1174 compared to the utilization of antibiotic or phage ST-3 alone. Additionally, this combination can inhibit the biofilm formation of *S. Typhimurium* CGMCC 1.1174.	Lu et al. (2022)

While these studies highlight the potential of single phage interventions, the inherent ability of bacteria to develop resistance to individual phages is a recognized challenge. This observation of emerging phage resistance, even if overcome by other specific phages, implicitly drives the field toward more robust strategies, such as phage cocktails and engineered phages, designed to preempt or manage this evolutionary pressure.

#### 2.2.2 Phage cocktails: broadening efficacy and mitigating resistance

To address the limitations of single phage therapy, particularly their narrow host range and the potential for rapid emergence of phage-resistant bacterial mutants, phage cocktails have gained prominence. Phage cocktails are formulations containing multiple distinct phages, often selected for their complementary lytic spectra or different mechanisms of action, thereby broadening the overall efficacy and significantly reducing the likelihood of resistance development (Monteiro et al., 2019). Yadav et al. orally administered a phage cocktail (phage hM1, hHC/T or HC-M1) to a group of albino mice infected with *S. Typhimurium* for 7 days. The results revealed that not only did the mice survive without any deaths but also that the bacteria were completely eradicated and remained absent for up to 2 months. In contrast, the untreated control group presented a 100% mortality rate. These findings indicate that phage cocktail treatment provides a high level of antimicrobial efficacy and has a prolonged effect (Yadav and Nath, 2022). In a separate study, a cocktail comprising five distinct phages (phages A7, A8, B3, A4 and A5) was evaluated on chicken meat contaminated with various *Salmonella* serotypes, resulting in a 1.4 Log_10_ reduction in bacterial load compared with the control (Aguilera et al., 2022).

#### 2.2.3 Microencapsulation and advanced delivery strategies

The therapeutic efficacy of bacteriophages can be significantly hampered by their susceptibility to harsh environmental conditions, such as acidic pH in the stomach, high temperatures during feed processing, or the presence of digestive enzymes (Colom et al., 2017). To overcome these challenges and enhance phage stability, delivery, and lytic activity, microencapsulation techniques have been developed. Microencapsulation involves entrapping phages within a protective matrix, which can shield them from detrimental conditions and facilitate their targeted release at the site of infection (Vinner and Malik, 2018). In a recent study, supplementation of the starting diet with L100-encapsulated phage as a feed additive during rearing significantly reduced the incidence of flock contamination with *S. Enteritidis*. Moreover, complete eradication of this pathogen from the environment was achieved, accompanied by a reduction in *Salmonella* colonization and excretion at the end of the rearing period (Lorenzo-Rebenaque et al., 2022). Parallel research confirms these findings, showing that microencapsulated phage T156 exhibits superior stability and enhanced lytic activity against drug-resistant *Salmonella* strains (Li et al., 2021).

#### 2.2.4 Phage-antibiotic synergy (PAS)

While phages are often considered alternatives to antibiotics, emerging evidence suggests that combining phages with conventional antibiotics can lead to synergistic effects, producing superior therapeutic outcomes compared to either agent used alone-a strategy known as Phage-Antibiotic Synergy (PAS) (Jo et al., 2016). This approach represents a paradigm shift from viewing phages solely as replacements for antibiotics to recognizing them as potentially valuable partners in combating bacterial infections, especially those caused by MDR strains. PAS offers multiple advantages, including enhanced bactericidal efficacy through complementary mechanisms of action, a reduced likelihood of resistance development to either agent, an expanded spectrum of activity against diverse serovars, and the potential for minimized adverse effects through dose reduction of both components (Strathdee et al., 2023; Threlfall, 2002). Several studies have demonstrated the benefits of PAS against *Salmonella*. The combination of phage KM16 with kanamycin sulfate exhibited better antimicrobial efficacy against *S. Paratyphi* A NA3 than kanamycin alone (Jiang et al., 2021). Similarly, Abdallah et al. reported phage ZCSE9 combined with kanamycin showed significantly higher antibacterial effects against multiple *Salmonella* serotypes, including *S. Typhi*, *S. Typhimurium*, and *S. Enteritidis*, compared to either agent used individually (Abdelsattar et al., 2023). Another study reported that phage phiPT1, when combined with subinhibitory concentrations of cefixime, gentamicin, ciprofloxacin, or aztreonam, resulted in significantly greater log reductions of *S. Enteritidis* PT1 compared to the antibiotics alone. The enhanced activity was attributed to antibiotic-induced changes in bacterial morphology (e.g., elongation/filamentation), which accelerated cell lysis by increasing sensitivity to lytic enzymes, increasing the burst size of phages, and upregulating receptor expression, thereby increasing phage adsorption rates (Alharbi et al., 2023). This detailed mechanistic understanding suggests that PAS is not merely an additive effect but a true synergy based on specific biological interactions, allowing for more rationally designed combinations.

Such findings underscore the potential of integrated phage-antibiotic therapies to enhance treatment efficacy, overcome resistance, and potentially “rescue” or extend the lifespan of existing antibiotics. However, it should be noted that these effects are highly context-dependent, and antagonistic interactions may occur under certain conditions, such as when antibiotics are used at bactericidal concentrations that fully suppress host replication machinery.

### 2.3 Phages and biofilm control

Phages also address another critical challenge in *Salmonella* infections - the formation of recalcitrant biofilms. Biofilms are complex, sessile microbial communities living on surfaces and interfaces where organisms produce a matrix of extracellular polymeric substances (EPS) (Flemming et al., 2023). This mode of growth provides bacteria, including *Salmonella*, with a protected microenvironment, significantly increasing their resistance to antibiotics and host immune defenses. The ability of phages to disrupt biofilms represents a critical advantage, extending their utility beyond acute planktonic infections to persistent, hard-to-treat biofilm-associated conditions (Yin et al., 2019; Roy et al., 2018). Rashad et al. demonstrated that *in vitro* applications of phage LPSent1 inhibited free planktonic cells and biofilms of *S. Enteritidis* EG. SmE1. Furthermore, this inhibition was associated with a reduced emergence of phage-resistant bacterial mutants (Al-Hindi et al., 2023; Geredew Kifelew et al., 2019). Consistent with this work, Yifeng et al. reported that treatment with phage T102 markedly inhibited biofilm formation and reduced the population of *S. Typhimurium* ATCC14028 (Ding et al., 2023). Additionally, Kwon et al. (2021) reported that the biomass decreased steeply within 6 h after *Salmonella* was treated with the high or low concentration phage pSal-SNUABM-02, demonstrating the antibacterial effects of the phage against *Salmonella*. At the same time, phages combined with antibiotics and phage cocktails have also been shown to be effective. As phages can disrupt biofilm structure, antibiotics more easily penetrate and reach bacteria. Min lu et al. reported that the combination of phage ST-3 with levofloxacin hydrochloride caused a significant decrease in biofilm biomass, which in turn contributed to a higher clearance rate of *S. Typhimurium* CGMCC 1.1174 than monotherapy. Katarzyna et al. reported that mixing phage cocktails vB-Sen-TO17 and vB-SenM-2 with 12.5 μg/mL tetracycline, 50 μg/mL colistin and enrofloxacin, respectively, enhanced the therapeutic efficacy of antibiotics on *Salmonella* and delayed the development of *Salmonella* resistance to antibiotics (Kosznik-Kwaśnicka et al., 2022). These findings underscore the potential of integrated phage-antibiotic therapies to enhance treatment efficacy, overcome resistance limitations, and optimize pathogen control strategies. Future research should explore mechanistic interactions and clinical scalability of such combinatorial approaches.

### 2.4 Engineered bacteriophages and phage-derived products

#### 2.4.1 Genetic engineering strategies (e.g., CRISPR-Cas, BRED) for enhanced phage therapeutics

While naturally occurring phages hold considerable promise, their therapeutic application can be limited by factors such as narrow host range and the potential for bacteria to develop resistance. To address these limitations and enhance therapeutic efficacy, phage engineering and the utilization of phage-derived lytic components have emerged as significant areas of research, representing a move toward “designer” biologicals tailored for specific needs (Mahler et al., 2023; Brödel et al., 2024).

Phage engineering, employing advanced gene editing techniques, offers a powerful means to modify phage genomes to improve their therapeutic properties. Key goals of phage engineering include expanding their host range to cover a wider array of pathogenic strains, increasing their lytic efficacy, and making them more resilient to bacterial defense mechanisms. Several sophisticated genetic engineering techniques are being applied, including conventional homologous recombination, the use of Lambda Red recombinases, Bacteriophage Recombineering of Electroporated DNA (BRED), various CRISPR-Cas systems (e.g., Cas9, Type III-A, Type VI), and Retron systems (Jia et al., 2023). These techniques allow for precise modifications to phage genomes. For example, phage host range can be modified by altering or swapping genes encoding receptor binding proteins (RBPs), which determine attachment to bacterial surfaces. Techniques like BRED improve DNA introduction for recombination, while CRISPR-Cas enables precise gene editing. These engineered phages can bypass bacterial resistance and target multiple *Salmonella* serovars, addressing limitations of natural phages and making phage therapy more reliable and widely applicable. Rational phage design is key to advancing the field (Jia et al., 2023).

#### 2.4.2 Endolysins: novel antimicrobials derived from phages

Endolysins are enzymes produced by bacteriophages during the late stages of their lytic cycle to degrade the bacterial cell wall from within, facilitating the release of progeny phages (Tung et al., 2025). When purified and applied externally, these enzymes can rapidly lyse bacteria, making them attractive as novel antimicrobial agents. Endolysins offer several potential advantages over whole phages and traditional antibiotics: they can exhibit high specificity for the peptidoglycan of target bacteria (though this is more pronounced for Gram-positive pathogens), act very rapidly, have a low propensity for inducing bacterial resistance, and can be effective against antibiotic-resistant strains and biofilms (Tung et al., 2025).

For Gram-negative bacteria like *Salmonella*, the outer membrane typically acts as a barrier to exogenously applied endolysins. Therefore, strategies such as co-administering endolysins with outer membrane permeabilizers (e.g., EDTA, organic acids) or engineering endolysins by fusing them with peptides that can disrupt the outer membrane are often necessary to achieve efficacy (Tung et al., 2025). Examples of endolysins and engineered endolysins showing activity against *Salmonella* include:

- ENDO-1252: Derived from *Salmonella* phage-1252, showed a 1.15 log reduction of *S. Enteritidis* when combined with EDTA (Tung et al., 2025).
- LysKpV475: From a *Klebsiella* phage, exhibited enhanced bacteriostatic effects against *S. Typhimurium* when combined with polymyxin B or phage phSE-5, and a 2-log reduction when immobilized in a pullulan matrix (Tung et al., 2025).
- Lys1S-L9P: An engineered fusion of endolysin Lys1S (from phage SPN1S) with a sensitizer peptide L9P, achieved 4–5 log reductions of *S. Enteritidis* and *S. Typhimurium*.
- Salmcide-p1: From *Salmonella* phage fmb-p1, showed broad bactericidal activity against Gram-negative bacteria when combined with EDTA, effectively suppressing *Salmonella* growth in skim milk.

Other endolysins like rLysJNwz, ST01 (CecA: ST01 fusion), LySP2, XFII, LysSTG2, LysSE24, LysWL59, LyS15S6, BSP16Lys, ABgp46, and Lys68 have also been investigated, often in combination with permeabilizers or other agents, for their anti-*Salmonella* activity in various contexts, including on contaminated food surfaces like eggs and lettuce, or in animal models (Tung et al., 2025). The development of endolysins as standalone therapeutics or as part of combination strategies represents an alternative modality derived from phage biology. Their nature as defined protein molecules may make them more amenable to traditional pharmaceutical development pathways, including recombinant production and quality control, compared to the complexities of live, self-replicating phage preparations. However, strategies like encapsulation may be needed to improve their stability and delivery *in vivo* (Soto Lopez et al., 2025).

### 2.5 Advantages, limitations, and future directions in phage therapy

With a growing body of research validating its efficacy, phage therapy is becoming a promising alternative for salmonellosis treatment, as evidenced by increasing clinical trials demonstrating its translational potential. Despite the advantages of phage therapy, several limitations and challenges remain. First, many natural phages have a narrow host range, meaning a specific phage may only be effective against a limited number of bacterial strains or serovars, necessitating careful matching or the use of phage cocktails to broaden coverage. Second, bacteria can develop resistance to phages, although this can sometimes be mitigated by using cocktails or isolating/engineering new phages. Third, there is a lack of large-scale, rigorously controlled clinical trials that meet stringent medical and regulatory standards, which are essential for confirming safety and efficacy. Fourth, large-scale phage production poses challenges in ensuring purity, stability, cost-effectiveness, and consistency-particularly for cocktails. Additionally, regulatory pathways for phage therapy are still evolving in many regions, creating uncertainty for developers.

Future directions focus on overcoming these barriers. There is an urgent need for robust pharmacokinetic and pharmacodynamic (PK/PD) studies to determine optimal dosing and administration routes and to better understand phage behavior in vivo. Engineering phages with broader host ranges, stronger lytic activity, and resistance to bacterial defense mechanisms is a key research priority. Standardizing phage cocktail formulations and improving delivery systems (e.g., microencapsulation) to protect phages and enhance targeted delivery are also critical. Beyond these technical and regulatory hurdles, the development of engineered phages introduces additional biosafety and ecological considerations. Engineering phages with broader host ranges or enhanced lytic activity may pose risks to beneficial commensal microbiota in the animal gut, and raises concerns regarding unintended environmental dissemination following release from treated hosts. To mitigate these risks, proposed strategies include the use of strictly lytic phages, genetic safeguards to limit phage persistence or horizontal gene transfer, controlled dosing and administration routes, and post-treatment environmental monitoring under robust regulatory oversight (Liu et al., 2022).

## 3 Probiotics in the management of salmonellosis

### 3.1 Mechanisms of probiotic action against *Salmonella*

Probiotics are live microorganisms that, when ingested in adequate amounts, can enhance the intestinal flora by modulating immune responses, activating immune regulatory functions, and inhibiting the growth of pathogens (Kim et al., 2019), which are used to improve the health of the host and provide health benefits to the host. Probiotic microorganisms are widely found in water, soil, plants, and animal foods (Suez et al., 2019). Probiotics employ a multifaceted arsenal of mechanisms to combat *Salmonella* infection, often acting simultaneously through direct antagonism and indirect host-mediated effects. This multi-pronged approach may offer more robust and resilient protection compared to therapies with a single mode of action.

#### 3.1.1 Competitive exclusion and nutrient competition

A primary mechanism by which probiotics exert their protective effect is through competitive exclusion. Upon administration, probiotic strains colonize the intestinal tract, occupying available niches and adhesion sites on the gut epithelium. This physical occupation makes it more difficult for *Salmonella* to attach and establish a foothold, a critical early step in its infection process. Beyond physical space, probiotics also compete with pathogens for essential nutrients present in the gut lumen. By consuming these limited resources, probiotics can create an environment less conducive to *Salmonella* proliferation. For example, the probiotic strain isolated by Upadhaya et al. (2016) inhibited *Salmonella* by producing beneficial bacteria. Podnar et al. eliminated *Bacillus subtilis* exopolysaccharides by deleting the EPSA operon. The role of EPSA-O polysaccharides in the regulation of the social outcome of bacillus-dependent competition between *Bacillus subtilis* and *S. Typhimurium* is important for competitor perception (Podnar et al., 2022). Experiments such as these are evidence that probiotics can produce antimicrobial substances (Zhang et al., 2022; Kowalska et al., 2020).

#### 3.1.2 Production of antimicrobial substances

Many probiotic strains actively produce a variety of antimicrobial substances that directly inhibit or kill *Salmonella*. These include organic acids such as lactic acid and acetic acid, which lower the intestinal pH, creating an unfavorable environment for acid-sensitive pathogens like *Salmonella*. The undissociated forms of these organic acids can also penetrate bacterial cells, dissociate internally, and disrupt cellular functions (Closs et al., 2025). Other antimicrobial compounds produced by probiotics include hydrogen peroxide and, significantly, bacteriocins-small, ribosomally synthesized antimicrobial peptides that can have potent and often specific activity against related bacterial species, including *Salmonella*. For instance, Lacticaseibacillus rhamnosus GG (LGG) has been shown to produce specific antimicrobial peptides (PN-1 to PN-5) with demonstrated anti-*Salmonella* activity. The cell-free supernatant of *Bacillus subtilis* PS-216, containing pks cluster-dependent polyketobacterenes, inhibits the growth and biofilm formation of *S. Typhimurium*. Furthermore Samiullah et al. added four kinds of probiotics and synthetic probiotics and reported that the number of *Salmonella* bacteria in the cecum was reduced and that the cecal flora was significantly altered (Khan and Chousalkar, 2020). Cirilo et al. reported that probiotic supplementation improved excreta quality, lowered ammonia concentrations, and lowered humidity. In conclusion, probiotics improved the growth performance of poultry without affecting the intestinal morphology, serum metabolites, or liver and kidney metabolites (Cirilo et al., 2023).

#### 3.1.3 Enhancement of intestinal barrier function

A healthy intestinal barrier is crucial for preventing the translocation of pathogens and their toxins from the gut lumen into the bloodstream (Chandrasekaran et al., 2024). Probiotics contribute to the maintenance and enhancement of this barrier function in several ways. As mentioned, the production of organic acids can lower the local pH. More directly, certain probiotic strains can stimulate the production of mucus by goblet cells and enhance the expression and proper localization of tight junction proteins, such as occludin and claudin-1, which seal the gaps between intestinal epithelial cells. For example, Lactiplantibacillus plantarum postbiotics increased the expression of occludin and claudin-1 in mice. Supplementation with LGG has been shown to increase villus height and the villus height to crypt depth ratio in the ileum of chickens, indicative of improved intestinal integrity and absorptive capacity (Closs et al., 2025).

#### 3.1.4 Modulation of host immune responses

**Figure 2:**
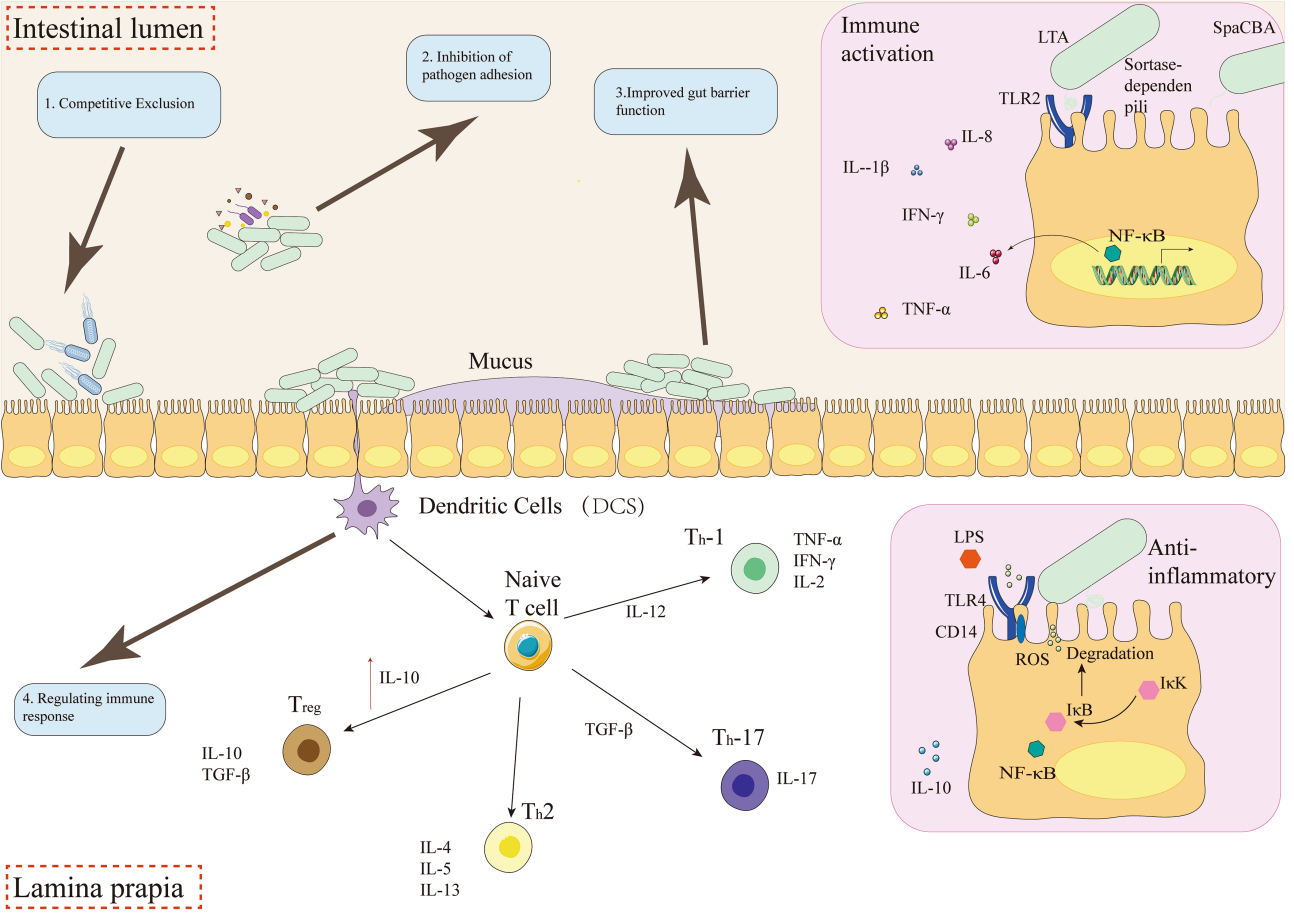
Probiotics, which are live beneficial bacteria, can be used to treat *Salmonella* infection. The mechanism of action can be divided into several stages: 1. Colonization of the intestine: Probiotics colonize the intestinal tract, competing for nutrients and space with the pathogen (*Salmonella*). 2. Competition with the pathogen: Probiotics outcompete *Salmonella* for adhesion and invasion sites, reducing the pathogen’s ability to colonize and cause disease. 3. Prevention of adhesion and invasion: Probiotics inhibit the adhesion and invasion of *Salmonella* into host cells, reducing the risk of disease. 4. Modulation of the immune response: Probiotics modulate the immune response by shifting the Th1/Th2 balance toward a Th2 response, which is associated with anti-inflammatory and protective effects. By targeting multiple stages of infection, probiotics can provide safe and effective treatments for *Salmonella* infection.

Probiotics can regulate immunity and reduce the expression of inflammatory factors. Huang and Huang (2016) evaluated the effects of two commonly used probiotics on *Salmonella*-induced IL-8 production in Caco-2 cells and their immunomodulatory function. Pradhan studied the probiotics LA and BC, *Lactobacillus acidophilus* (LA) and *Clostridium perfringens* (BC) and reported that they could inhibit the expression of inflammatory factors, such as Ccl17, Ccl24, Ccrl2, and CXCL12, and interleukins, such as IL 2, IL 6, IL 22, IL 22 ra1, and IL 24 (Pradhan et al., 2019) (Table 2; Figure 2). Moreover, they can interact with various immune cells, including epithelial cells, dendritic cells, macrophages, and lymphocytes, to orchestrate an immune response that is more effective against *Salmonella* while minimizing excessive inflammation. Mechanisms include shifting the Th1/Th2 cytokine balance, typically toward a Th2 anti-inflammatory response or a balanced Th1 response appropriate for clearing intracellular pathogens. Probiotics can reduce the production of pro-inflammatory cytokines such as IL-6, TNF-*α*, IL-1β, and IL-8, while simultaneously increasing the levels of anti-inflammatory cytokines like IL-10 and IL-4.1 For example, *Lactobacillus crispatus* 7–4, *Lactobacillus johnsonii* 3–1, and *Pediococcus acidilactici* 20–1 reduced inflammatory damage and pro-inflammatory cytokine expression while inducing IL-10 in *S. Enteritidis* infected models. Some probiotics, like *L. plantarum* RS-09, can modulate macrophage polarization toward an M1 (pro-inflammatory, pathogen-killing) phenotype and induce TLR2-associated NF-κB signaling activity against *S. Typhimurium.* Furthermore, probiotics can regulate the NOD-like receptor thermal protein domain-associated protein 3 (NLRP3) inflammasome, a key component of the innate immune response that, if overactivated, can contribute to inflammatory damage. Limosilactobacillus reuteri KUB-AC5 has also been shown to attenuate gut inflammation during *Salmonella* infection (Buddhasiri et al., 2021).

**Table 2:** Application of probiotic preparations in the treatment of *Salmonella*.

*Salmonella* serotypes	Probiotics	Function	Functional description	References
*S. Typhimurium*	*Lactobacillus Acidophilus* (LA) and *Bacillus clausii* (BC)	Immunomodulatory effect, Reducing the expression of inflammatory factors	LA can effectively improve the microbial dysbiosis and inflammation caused by *Salmonella* infection in Th1 (C57BL/6) and Th2 (BALB/c) biased mice. BC can improve the microbial dysbiosis and inflammation caused by *Salmonella* in Th 2.	Pradhan et al. (2019)
*S. Typhimurium* strains SL1344	*L. rhamnosus* GG (LGG) (ATCC 53103) *B. animalis* subsp. lactis (BL) (DSM 10140)	Immunomodulatory effect, Reducing the expression of inflammatory factors	Have significantly different (inhibitory or enhanced) effects on IL-8 response through the PI3K/Akt signaling pathway or the expression of NOD2 protein on the membrane.	Huang and Huang (2016)
*S. Enteritidis*	*Lactobacillus crispatus* 7–4, Lactobacillus johnsonii 3–1 and Pediococcusacidilactici 20–1	Immunomodulatory effect, Reducing the expression of inflammatory factors	Reduces inflammatory damage in the gut and liver, decreases the expression of pro-inflammatory cytokines (IL-6, TNF-α, IL-1β) and induces the expression of anti-inflammatory cytokines (IL-10)	Wu et al. (2023)
*Salmonella* gallinarum KVCC BA 0700722	*Bacillus subtilis* RX7 *B. methylotrophicus* C14	Produce antibacterial substances, Competitive Exclusion	Enzymes or antimicrobial agents produced by Bacillus; More Lactobacillus produces less *Salmonella*	Upadhaya et al. (2016)
*S. Typhimurium* CMCC 50115	Latilactobacillus curvatus (FY1), *Weissella cibaria* (FY2), Limosilactobacillus mucosae (FY3), and Lactiplantibacillus pentosus (FY4)	Produce antibacterial substances, Competitive Exclusion	FY4 exhibits adhesion capacity, high hydrophobicity, strong self-aggregation and coaggregation. Stimulates the host immune system to competitively reject pathogens and produce inhibitory metabolites against unfavorable microorganisms through antagonistic binding sites and nutrients	Zhang et al. (2022)
*S. Typhimurium* SL1344	*B. subtilis* PS-216	Produce antibacterial substances, Competitive Exclusion	*Bacillus subtilis* PS-216 inhibits the growth and biofilm formation of *S. Typhimurium* by producing pks cluster-dependent polyketobacterenes.	Podnar et al. (2022)
*S. Enteritidis* *S. Bongori*	Lactobacillus sp. strains: L.rhamnosus LOCK 1131, L.casei LOCK 1132, and *L. paracasei* LOCK 1133	Produce antibacterial substances, Competitive Exclusion	Stimulation of the immune system and prevention of pathogen colonization through the antagonistic activity of probiotics against *Salmonella* or their competition for a limited number of receptors on the surface of the GIT	Kowalska et al. (2020)
*S. Typhimurium*	Four commercial probiotic and synbiotic products	Protecting the intestinal barrier	Bifidobacterium protects intestinal bacteria against *Salmonella Typhimurium* infection	Khan and Chousalkar (2020)
S. Heidelberg	*B. amyloliquefaciens* *B. subtilis* *B. thuringiensis* *S. cerevisiae*	Protecting the intestinal barrier	Increased probiotic retention in the gastrointestinal tract by intestinal epithelial adhesion; *Bacillus subtilis* reduces NH3 emissions from poultry due to increased endogenous enzyme activity and nitrogen utilization	Cirilo et al. (2023)
*S. Enteritidis* *S. Typhimurium*	Ligilactobacillus salivarius 7247 (LS7247) s	Tolerance of probiotics to gastric and intestinal stresses	Lactic acid exerts its antimicrobial effect by targeting the bacterial cell wall, cytoplasmic membrane and specific metabolic functions of pathogen replication and protein synthesis, ultimately leading to pathogen destruction and death	Abramov et al. (2023)

The beneficial effects of probiotics are, however, highly strain-specific, and even species-specific. This implies that generic claims about “probiotics” are insufficient for therapeutic guidance; research must focus on meticulously characterizing and validating specific strains for their anti-*Salmonella* activity and mechanisms of action.

### 3.2 Key probiotic strains and their efficacy

Chen Pei et al. stimulated mouse monocyte macrophages with *Lactobacillus plantarum RS-9*. *Lactobacillus plantarum* RS-09 can reduce splenomegaly caused by *S. Typhimurium*, and *Lactobacillus plantarum* can interact with macrophages to prevent bacterial enteropathy because of its ability to modulate macrophage polarization, upregulate the generation of M1 macrophages and induce TLR2-associated NF-κB signaling activity against *S. Typhimurium* (Zhao et al., 2022). In 2023, Aixin et al. studied *Lactobacillus plantarum* postbiotics. Turbidimetry and agar diffusion experiments revealed that *Lactiplantibacillus plantarum*-derived postbiotics (LPC) directly reduced *Salmonella* growth. Real-time PCR and biofilm inhibition assays revealed that LPC strongly suppressed *Salmonella* pathogenicity by reducing the expression of virulence genes (SopE, SopB, InvA, InvF, SipB, HilA, SipA and SopD2), pili genes (FilF, SefA, LpfA, FimF), flagellum genes (FlhD, FliC, FliD) and biofilm formation genes. LP probiotics were more effective than LPs in attenuating ST-induced intestinal damage in mice, as indicated by increased villus/crypt ratios and increased expression levels of tight junction proteins (Occludin and Claudin-1).

### 3.3 Multistrain probiotics, synbiotics and postbiotics

The concept of using multistrain probiotic formulations is based on the rationale that a combination of different probiotic strains, potentially with complementary mechanisms of action, may offer enhanced protective effects compared to single-strain probiotics. This approach mirrors the strategy behind phage cocktails, aiming for broader efficacy and resilience. Chihua et al. demonstrated that multistrain probiotics have a better protective effect against *Salmonella* infection and concluded that compound probiotics are better than single-strain probiotics (Chang et al., 2020). Probiotics and medicines can also be used together to lower *S. Typhimurium* expression. Zul’s use of the combination of probiotics and Ajwain extract (which has the effects of clearing bacterial infections, improving oxidation, and inhibiting inflammation) proves this point of view (Haq et al., 2022). Huixianwu et al. further completed a simulation experiment involving the combination of probiotics on the basis of the finding that three probiotics can attenuate *Salmonella*. Microencapsulation technology is used to increase the stability and survival rate of probiotics. These findings indicate that microencapsulated probiotics are superior to free probiotics (Upadhaya et al., 2016).

The term “synbiotics” refers to combinations of probiotics and prebiotics (non-digestible food ingredients that selectively stimulate the growth and/or activity of beneficial bacteria in the colon). While the original manuscript mentions synbiotics in the context of protecting the intestinal barrier against *S. Typhimurium* infection, detailed exploration of synbiotic formulations specifically for *Salmonella* control is an area for further research. The exploration of multistrain probiotics and their combination with other beneficial agents reflects a broader principle in biotherapeutics: combining agents with diverse yet complementary mechanisms can lead to enhanced overall efficacy and a more robust therapeutic outcome.

Postbiotics are emerging as a significant evolution in the field of microbe-based therapies (Żółkiewicz et al., 2020). Defined as a “preparation of inanimate microorganisms and/or their components that confers a health benefit on the host”postbiotics include non-viable bacterial cells, cell fractions (like cell wall components, teichoic acids), and metabolites secreted by live bacteria (such as short-chain fatty acids (SCFAs), enzymes, peptides, and organic acids) (Cortés-Martín et al., 2020). The mechanisms of action of postbiotics are diverse and overlap with those of live probiotics. They can modulate the gut environment by reducing intestinal pH, enhance epithelial barrier functions, regulate local and systemic immune responses (often by upregulating anti-inflammatory cytokines and downregulating pro-inflammatory signals), and exert direct antimicrobial activity (Wuethrich et al., 2021). For instance, SCFAs, key postbiotic metabolites, serve as energy for colonocytes and can inhibit pathogen growth (Nataraj et al., 2020).

A compelling example is the use of LPC. LP postbiotics significantly reduced ST-induced inflammation by regulating the levels of inflammatory cytokines (increased IL-4 and IL-10 and decreased TNF-*α*) (Hu et al., 2023). Furthermore, LP postbiotics inhibited the activation of the NOD-like receptor thermal protein domain-associated protein 3 (NLRP3) inflammasome by decreasing the protein expression of NLRP3 and Caspase-1 and the gene expression of Caspase-1, IL-1β, and IL-18. Moreover, both LPC and LPB noticeably activated autophagy during ST infection, as indicated by the upregulated expression of LC3 and Beclin1 and the downregulated p62 level (*p* < 0.05). Finally, we found that LP postbiotics could trigger the AMP-activated protein kinase (AMPK) signaling pathway to induce autophagy.

Postbiotics offer several distinct advantages over live probiotics, directly addressing many of their practical limitations. These include enhanced stability (less sensitive to heat, oxygen, humidity, and gastric acidity), a superior safety profile (no risk of administering live microbes, especially crucial for immunocompromised individuals, children, or premature neonates), longer shelf-life, and potentially easier standardization, storage, and transport. These characteristics make postbiotics highly attractive for broader clinical and commercial development. Furthermore, research into postbiotics helps to deconstruct the beneficial effects of live probiotics by identifying the specific bioactive molecules responsible. This allows for a more precise, mechanism-based therapeutic development, moving from using the “whole organism” to utilizing its defined “active ingredients,” which aligns more closely with traditional pharmacological approaches.

### 3.4 Next-Generation Probiotics (NGPs) and engineered strains

The field of probiotic research is evolving beyond traditional strains, with increasing interest in “Next-Generation Probiotics” (NGPs). NGPs are novel microbial strains, often commensals isolated from the human gut, that exhibit specific health benefits and are typically developed with a pharmaceutical application in mind, sometimes referred to as Live Biotherapeutic Products (LBPs) (Li et al., 2024). These represent a shift toward more targeted and potentially more potent interventions. An example of an NGP with potential relevance is *Akkermansia muciniphila*. This Gram-negative bacterium colonizes the mucus layer of the human gut and is considered a promising NGP due to its various beneficial effects, including enhancing intestinal barrier integrity and modulating host immune responses, which could indirectly help protect against pathogens like *Salmonella* (Li et al., 2024; Ma et al., 2022). Furthermore, Micromycin produced by Sassone-Corsi’s *E. coli* Nissle 1917 strain (EcN) can displace pathogenic Enterobacteriaceae species under specific environmental conditions, demonstrating that MccH47 can inhibit the growth of *Salmonella in vitro* (Sassone-Corsi et al., 2016). Therefore, Jacob developed the probiotic MccH47 with an induction system (an ECN-engineered strain containing a plasmid system of mchAXIBCDEF and ttrRSBCA). The EcN-engineered strain carries a plasmid system that inhibits the ability of *Salmonella* to grow when exposed to tetrasulfate, a molecule produced in the inflamed gut during *Salmonella* infection. MccH47 is created under the action of tetra sulfate, verifying the suppression and competition of *Salmonella in in vitro* tests (Palmer et al., 2018). In the future, *in vivo* simulation experiments will be used to achieve stable chromosomal integration, and the results will be subsequently applied to the clinical trial stage. In the near future, this genetically designed probiotic medicine will play an important role in clinical practice.

### 3.5 Advantages, limitations, and future directions in probiotic therapy

While probiotics show promise in combating *Salmonella* infections, their clinical application faces several challenges:1. Stability and Storage Constraints. Many probiotic strains are sensitive to heat, oxygen, and humidity. Exposure to temperatures above 25 °C during storage or transport can significantly reduce viability, compromising therapeutic efficacy. 2. Variable Therapeutic Outcomes. The gut microbiota composition, immune status, age, and genetic background of the host influence probiotic colonization and efficacy. In addition, the inherent stability and colonization resistance of the resident gut microbiota often limit the persistence and functional impact of administered probiotics, contributing to substantial inter-individual variability in therapeutic outcomes. 3. *Salmonella*-induced gut inflammation may alter the mucosal environment, hindering probiotic adhesion and persistence. 4. Chronic probiotic use might disrupt indigenous flora balance or delay post-infection microbiome recovery. 5. Lack of Clinical Protocols: Limited large-scale human trials hinder consensus on treatment duration, strain combinations, or adjuvant therapies. 6. High-quality control requirements and cold-chain logistics increase costs, limiting accessibility in resource-limited regions.

Future research in probiotic therapy should focus on overcoming these limitations. Key priorities include the development of engineered probiotics with enhanced characteristics, such as improved acid and bile resistance for better survival in the gastrointestinal tract, and targeted antimicrobial properties for greater efficacy against specific pathogens like *Salmonella*. As discussed previously, exploring postbiotic alternatives, such as heat-killed probiotics (paraprobiotics) and specific metabolite-based therapies (e.g., LP postbiotics), represents a highly promising avenue. These non-viable preparations could circumvent the challenges associated with the stability and viability of live probiotic formulations, offering more reliable and potentially safer strategies for combating *Salmonella* infections. Similar to phage therapy, a key overarching challenge for probiotics is to ensure consistent efficacy and overcome practical issues of viability and delivery to translate their in vitro and preclinical promise into reliable clinical outcomes and widely available commercial products. The increasing focus on postbiotics is a direct response to these viability-related challenges.

## 4 Vaccines for the prevention and control of salmonellosis

### 4.1 Overview of vaccine strategies: inactivated (killed), live-attenuated, and subunit vaccines

As a cornerstone of One Health approaches, vaccination represents the most sustainable intervention for controlling *Salmonella* transmission across human-animal-environment interfaces (Adams et al., 2011). Three principal vaccine platforms have been developed against *Salmonella*: inactivated (killed) vaccines, attenuated vaccines, and subunit vaccines. While inactivated vaccines reliably induce humoral immunity through antibody production, their inability to stimulate robust cellular immune responses limits long-term protection. In contrast, attenuated vaccines-developed through precise genetic modifications to reduce virulence while maintaining immunogenicity-offer distinct advantages:1. they mimic natural infection to elicit both mucosal IgA and systemic IgG responses, 2. they activate CD4 + and CD8 + T-cell immunity critical for intracellular pathogen clearance, and 3. they often require fewer doses due to their capacity for limited replication in host tissues (Chen et al., 2021). These superior immunogenic properties, combined with practical advantages in administration, have positioned attenuated vaccines as the preferred choice for both human and veterinary applications (Table 3).

**Table 3:** Overview of vaccine strategies against *Salmonella*, including inactivated, live-attenuated, and subunit vaccines.

*Salmonella* serotypes	Vaccine type	Route of administration	Function	Functional description	References
*S. Enteritidis* *S. Typhimurium*	Attenuated vaccine	Subcutaneous injection, Oral	Produce specific immune responses	Presents all its antigens throughout the body. Laying hens infected with invasive *Salmonella* strains have significantly improved immunity to reinfection.	Penha Filho et al. (2010)
*S. Enteritidis*	Attenuated vaccine	Oral	Activate the immune system, produce a specific immune response, preventive effects.	Construction of a sptP deletion mutant C50336ΔsptP in *S. Enteritidis*.	Lin et al. (2017)
*S. Enteritidis*	Attenuated vaccine	intramuscular injection	Produce specific immune responses.	Immunization with the mutant induced highly specific humoral immune responses and the expression of cytokines IFN-γ, IL-1β, and IL-6.	Li et al. (2019)
*S. Enteritidis*	Attenuated vaccine	Oral	Activate the immune system, produce specific immune responses, and have preventive effects.	Dual protection mechanism by increasing mucosal immunity and generating specific bacterial competition against any wild-type *Salmonella*	Huberman et al. (2019)
*S. Enteritidis*	Subunit vaccines	intramuscular injection	Activate the immune system, produce specific immune responses, and have preventive effects.	Recombinant rHis-SseB and rGST-SseB proteins were expressed in *E. coli* and confirmed by Western blotting to be immunoreactive with antiserum from *S. Enteritidis* C50041.	Kang et al. (2022)
*S. Enteritidis*	Inactivated vaccine	intramuscular injection	Produce specific immune responses	Vaccine induces protective immunity against MG One or more purified antigens, killed pathogens, or bacteria with oil adjuvants can successfully induce immune responses.	Marouf et al. (2022)
*S. Enteritidis* *S. Typhimurium*	Attenuated vaccine	intramuscular injection	Vaccines inhibit pathogen activity and have a preventive effect	Vaccines with consistent antimicrobial activity	Lin et al. (2022)
*S. Enteritidis*	Attenuated vaccine	Oral	Activate the immune system, produce specific immune responses, and have preventive effects.	IL-1β, IL-6, IL-12β, and TNF-α exert pro-inflammatory properties and innate immune activity. IL-10 inhibits cytokine production and monocyte function and has anti-inflammatory effects	Jan et al. (2023)
*S. Enteritidis* *S. Typhimurium*	Attenuated vaccine	Oral	Stimulate the immune system to produce specific immunity	*Salmonella-*specific antibodies (IgG and IgA) and microbiota structure, by comparing the expression of cytokines associated with type 1 (IL-18, TNF-α), type 2 (IL-4, IL-10, IL-6), and type 3 (IL-17A, IL-8) immune responses	Lyimu et al. (2023)

#### 4.1.1 Attenuated vaccines

When fed orally, live-attenuated *Salmonella* strains induce secretory, humoral, and cellular anti-*Salmonella* responses in the host and are potent oral immunogens (Chatfield et al., 1989). Zhi Jie generated the *Salmonella* protein tyrosine phosphatase (sptP) gene deletion mutant strain C50336ΔsptP in *S. Enteritidis* C50336 through *λ*-Red-mediated recombination, which also inhibited the production of interleukin-8 (IL-8) and inhibited the host inflammatory response, thereby promoting *Salmonella* invasion and intracellular replication (Lin et al., 2017). ChenSi evaluated AviPro *Salmonella* DUO, a bivalent live attenuated vaccine containing the *S. Enteritidis* strain Sm24/ Rif12/ Ssq and the *S. Typhimurium* strain Nal2/ Rif9/ Rtt, and discovered that a single dose of the vaccine inhibited *salmonella*. *Salmonella* shedding and tissue invasion were effectively and considerably reduced following the three dosages (Lin et al., 2022). Jan also studied the mechanism of the attenuated *Salmonella* live vaccine AviPro *Salmonella* Duo against *S. Enteritidis* infection. He conducted cytokine mRNA expression and 16S rRNA metagenomic analysis and reported that *S. Enteritidis* challenges significantly upregulated expressions of IFNg, IL-1β, IL-12β, and NFκB1A in SPF layers hens. Specifically, the vaccine significantly counteracted the levels of IFNa, IFNg, and NFκB1A activated by *S. Enteritidis* challenge. When complete immunity is achieved through three vaccines, there is no bacterial shedding, and the *Salmonella* burden in tissues substantially decreases (Jan et al., 2023). Qiuchun Li constructed two candidate vaccines, CZ14-1DspiCDnmpC and CZ14-1DspiCDrfaL. Immunity with the candidate mutants induced highly specific humoral immune responses and the expression of the cytokines IFN-*γ*, IL-1β, and IL-6. The results demonstrated that these two vaccination mutant strains can grow into live attenuated vaccines (Li et al., 2019). The above-attenuated vaccines all have strong protective effects, and live attenuated vaccines are better at stimulating the body’s immune response. However, the safety of live attenuated vaccines in the body, as well as the possibility of mutations, make widespread usage unlikely. For unstable factors, much research still needs to be conducted, and if a preventive vaccine is to be developed, it may be able to prevent only one serotype (Sabag-Daigle et al., 2016). Although inactivated vaccines can stimulate the production of antibodies, they are not effective at increasing the proliferation of immune system cells. Inactivated vaccines also have numerous drawbacks, including being swiftly removed by the host and failing to elicit cellular immune responses (Olszewska and Steward, 2001).

#### 4.1.2 Subunit vaccines

Subunit vaccines, which are made up of defined antigens, are safe and simple to administer, and they provide a novel approach to salmonellosis prevention. XiLong developed an *S. Enteritidis* subunit vaccine candidate based on rHis-SseB, with simvastatin used as an adjuvant to enhance SseB-specific humoral and cellular immune responses. Combined immunization with rHis-SseB and simvastatin conferred protective immunity against *S. Enteritidis* infection and significantly reduced bacterial colonization in target organs (Kang et al., 2022).

### 4.2 Novel platforms: mRNA vaccines and viral vectors

The remarkable success of mRNA vaccine technology during the COVID-19 pandemic has spurred interest in its application to other infectious diseases, including bacterial infections like salmonellosis. mRNA vaccines work by delivering genetic instructions (in the form of mRNA encoding specific antigens) to host cells, which then produce the antigen, triggering an immune response. This platform offers potential for rapid development and adaptation, as antigen sequences can be quickly designed and synthesized. While research into mRNA vaccines for *Salmonella* is still in its early stages, it represents a promising new frontier (Ramachandran et al., 2022). The rapid technology transfer from virology to bacteriology highlights the adaptability of this platform. Viral vectors, where genes encoding *Salmonella* antigens are inserted into harmless viruses, are another strategy, although less prominently featured for *Salmonella* in recent snippets compared to mRNA or direct S*almonella*-based vectors for multivalent vaccines.

### 4.3 Nanoparticle-based delivery systems for enhanced immunogenicity

Nanoparticle (NP)-based delivery systems are emerging as a powerful tool to enhance the efficacy of vaccines, particularly subunit and oral vaccines, against pathogens like *Salmonella*. NPs can protect vaccine antigens from degradation (e.g., in the harsh environment of the gastrointestinal tract for oral vaccines), improve their stability, facilitate controlled or sustained release (creating a “depot effect”), enhance antigen uptake and presentation by antigen-presenting cells (APCs), and act as adjuvants themselves to boost immunogenicity (Wang et al., 2023). Chitosan nanoparticles (CNPs) have been investigated for oral delivery of *Salmonella* antigens in broilers. Studies have shown that CNP-encapsulated *Salmonella* vaccines can reduce gut permeability changes induced by infection, decrease *S. Enteritidis* load in the ceca, spleen, and small intestine, and increase levels of antigen-specific IgY and IgA. The positive surface charge of chitosan NPs facilitates adherence to negatively charged mucosal surfaces, aiding antigen delivery to mucosal immune inductive sites. Polymeric nanoparticles, in general, can be tailored in size (typically 10–500 nm) to optimize uptake by APCs and can be designed for pH-responsive release of antigens (Acevedo-Villanueva et al., 2021). Nanoparticle delivery systems are particularly crucial for developing effective oral killed vaccines, as they address the major hurdle of antigen degradation in the GI tract, thereby enabling efficient mucosal immune stimulation (Wang et al., 2023; Wang et al., 2023).

### 4.4 Clinical application of *Salmonella* vaccines

While preclinical research on novel *Salmonella* vaccines is vibrant, the translation to human clinical trials, particularly for NTS, is an ongoing process. Existing clinical trial data, as summarized in Table 4, predominantly focuses on vaccines against typhoidal *Salmonella* strains, such as *S. Typhi* and *S. Paratyphi*. These trials have evaluated various candidates, including the Typhoid Vi polysaccharide vaccine, Vi-CRM197 conjugate vaccine, the live attenuated M01ZH09 (*S. Typhi* Ty2 ΔaroC ΔssaV), and the live oral attenuated CVD 1902 for *S. Paratyphi* A. These trials have assessed immunogenicity (e.g., antibody responses) and safety in diverse populations, including adults and children in various geographical regions.

**Table 4:** Clinical application of *Salmonella* vaccines.

Start and end time	Area	Project	Vaccine	Research purposes	Completion rate	NCT
2012–2014	Japan	Study of a single dose of SP093 Typhoid VI polysaccharide vaccine in Japanese subjects	Typhoid Vi polysaccharide vaccine (intramuscular injection)	Immunogenicity and safety of single-dose SP093 Typhoid VI polysaccharide vaccine	100%	NCT01608815
2011–2014	Philippines	Safety and immunogenicity of Vi-CRM197 vaccine against *Salmonella typhi* in children, older infants, and infants	Vi-CRM197 vaccine pneumococcal conjugate vaccine Vi polysaccharide (PS) vaccine	Safety, reactogenicity, and immunogenicity of NVGH glycoconjugate vaccine against *Salmonella typhi* in children, older infants, and infants	99.16%	NCT01437267
2010–2014	India, Pakistan	Safety and immunogenicity of Vi-CRM197 vaccine in adults, children, older infants, and infants	Vi-CRM197 vaccine pneumococcal conjugate vaccine Vi polysaccharide (PS) vaccine	Safety, reactogenicity, and immunogenicity of NVGH glycoconjugate vaccine against *Salmonella typhi* in adults, children, older infants, and infants	93.88%	NCT01229176
2011–2014	Germany	A Phase 3b, randomized, open-label study to evaluate the safety and immunogenicity of a specific travel vaccine when administered concurrently with MenACWY in adults	Typhoid VI polysaccharide vaccine yellow fever vaccine Japanese encephalitis vaccine	Safety and immunogenicity of the vaccine when administered concomitantly with the Novartis meningococcal ACWY conjugate vaccine in healthy adults	99.09%	NCT01466387
2006–2012	Vietnam	Safety, immunogenicity, and compatibility of infant typhoid vaccine with DTP	Vi-rEPA typhoid conjugate vaccine Hib-TT	Safety, immunogenicity, and compatibility with DTP of an investigational Vi-rEPA conjugate vaccine administered concomitantly with DTP in Vietnamese infants	80.06%	NCT00342628
2010–2013	Belgium	Safety and immunogenicity of Vi-CRM197 vaccine against *Salmonella typhi* in adults (18–40 years)	Typhus NVGHVi-CRM197	To evaluate the safety and immunogenicity of the NVGH glycoconjugate vaccine against *Salmonella typhi* in adult subjects aged 18 to 40 years.	94%	NCT01123941
2008–2017	America	Immunogenicity, safety, and tolerability of typhoid vaccine candidate M01ZH09 in healthy adults	M01ZH09	Determining the immunogenicity, safety, and tolerability of *Salmonella typhi* (Ty2 are-ssaV-) ZH9,	97.86%	NCT00679172
2010–2013	Belgium	Safety and immunogenicity of three formulations of Vi-CRM197 vaccine against *Salmonella typhi* in adults (18–40 years)	NVGH Vi-CRM197 Vi-polysaccharide vaccine	Evaluating the safety and immunogenicity profiles of three Vi-CRM197 conjugate vaccines against *Salmonella typhi* compared with currently licensed Vi polysaccharide vaccines in healthy adults	97.72%	NCT01193907
2011–2014	Belgium	H04_197TP extension study to assess enhanced responses induced by Vi-CRM01 in adults	NVGH Vi-CRM197	To evaluate the enhanced response induced by Vi-CRM197 following priming with Vi-CRM197 or Typherix in adult subjects H01_04TP Study	90.19%	NCT01438996
2010	America	Safety and immunogenicity of CVD 1902 oral attenuated vaccine to prevent Streptococcus paratyphi A infection	CVD 1902, a live oral vaccine against *Salmonella enterica* Serovar Paratyphi A	To determine whether CVD 1902, a live attenuated oral vaccine, is safe and effective in preventing *S. enterica* serovar Paratyphi A infection.	NA	NCT01129453
2014	Britain	*Salmonella Typhi* Vaccine (VAST)	Vi-TCVVi-PS vaccine Control group (men ACWY)	To evaluate the immunogenicity and protective efficacy of Vi-conjugated (Vi-TCV) and unconjugated (Vi-PS) polysaccharide vaccines compared with a control vaccine (meningococcal ACWY) in preventing typhoid infection using a human typhoid infection challenge model	NA	NCT02324751
2022	America	*Salmonella* CVD 2000 Conjugate: Study of Vaccination Response to Trivalent *Salmonella* Conjugate Vaccine for Prevention of Invasive Salmonellosis	Trivalent *Salmonella* Conjugate Vaccine (TSCV)	Comparison of the safety, reactogenicity, and immunogenicity of trivalent *Salmonella* (*S. enteritidis*/*S. typhimurium*/*S. typhimuriumvi*) conjugate vaccine (TSCV) full-strength formulations, half-strength formulations of TSCV, and diluted half-strength doses of TSCV against invasion salmonellosis,	NA	NCT05525546
2019	America	*Salmonella* conjugate CVD 1000: a study of vaccination responses to trivalent invasive salmonellosis vaccine.	Trivalent invasive salmonellosis vaccine	Safety, reactogenicity, and immunogenicity of a trivalent conjugate vaccine against invasive salmonellosis.	NA	NCT03981952
2009–2011	America	Recombinant attenuated *Salmonella typhi* vaccine vector producing *Streptococcus pneumoniae* PspA	*Salmonella Typhi*-Vector Pneumonia Vaccine RASV Strain	Phase I comparison of the safety and immunogenicity of three recombinant *Salmonella typhi* vaccine vectors producing the *Streptococcus pneumoniae* surface protein antigen PspA in adult volunteers	NA	NCT01033409

### 4.5 Combination vaccines

As vaccine research advances, experts have learned that they can offset the weaknesses of vaccines when they are used in combination. Research has evaluated protein formulations as vaccine candidates, comparing their immune response, protection, and organ clearance. The results showed that the recombinant protein had good immunogenicity. These findings indicate that the protein preparation has a protective effect on *Salmonella* infection and that the combination of RBapB, Rompa, and ROmpC has a good protective effect and organ clearance effect on the challenge of virulent *S. Typhimurium* and *S. Enteritidis.*

To address the issue of drug resistance induced by antibiotics used during the early stages of salmonellosis treatment. Jiangang determined that the *S. Enteritidis* live vaccine (Sm24/Rif12/SSQ strain) can prevent *S. Enteritidis* infection and studied whether the use of antibiotics affects the colonization of the oral live vaccine. The ecological balance of intestinal microorganisms is disrupted by oral or intramuscular injection of antibiotics and then vaccination with *Salmonella* (Sm24/Rif12/SSQ strain). Live attenuated *Salmonella* vaccine strains were finally isolated (Huberman et al., 2019).

In addition, attenuated strains obtained through genetic engineering that are safe but still immunogenic can be further engineered by introducing additional coding; the resulting multivalent vaccine candidates should be able to challenge the vector vaccine itself as well as additional target pathogens with biologically relevant protective immune responses (Galen et al., 2021).

### 4.6 Advantages, limitations, and future directions in vaccine development

Vaccines offer numerous advantages, including the potential to confer long-term immunity, prevent primary infection, reduce pathogen shedding, and contribute to the development of herd immunity within a population. However, the development of *Salmonella* vaccines is not without limitations. As mentioned above, the serotype specificity of many traditional vaccines is a major obstacle for NTS. Safety concerns, as well as the costs of vaccine development, production, and deployment, can be substantial, particularly for novel vaccine platforms. Some vaccines may require multiple injections or booster doses to achieve and maintain protective immunity, and adverse reactions may occur.

Future directions in *Salmonella* vaccine research are aimed at overcoming these limitations. A primary goal is the development of broadly protective NTS vaccines, leveraging multivalent antigen strategies, reverse vaccinology to identify conserved epitopes, and novel platforms like bacterial ghosts. Advancing mRNA and nanoparticle-based delivery systems for *Salmonella* antigens holds promise for improved immunogenicity and potentially faster development timelines. The development of more effective and safer adjuvants, particularly for subunit and mucosal vaccines, is crucial. Oral vaccine formulations that can efficiently induce robust mucosal immunity are highly desirable for enteric pathogens. Additionally, the development of DIVA (Differentiating Infected from Vaccinated Animals) vaccines is important for disease surveillance and control programs in livestock. Ultimately, achieving a broadly protective, safe, and cost-effective NTS vaccine remains a major objective in *Salmonella* research, the realization of which would have a profound impact on global food safety and public health.

## 5 Combination biotherapies: synergistic approaches to combat

The limitations inherent in single biotherapeutic approaches, such as the narrow host range of some phages or the variable efficacy of certain probiotics, have spurred research into combination therapies. The rationale is that by combining different biological agents with distinct but complementary mechanisms of action, it may be possible to achieve synergistic effects, leading to enhanced efficacy, broader protection, and a reduced likelihood of resistance development.

### 5.1 Phage-probiotic combinations

Recent studies have explored the combined use of bacteriophages and probiotics as a synergistic strategy to enhance anti-*Salmonella* efficacy while mitigating limitations associated with single-modality treatments. In this context, *Lactobacillus plantarum* with phage resistance was cultured in 2019, and the LP^+PR^ strain significantly inhibited the adhesion and invasion abilities of INT-407 cells (*p* < 0.05) (Nagarajan et al., 2019). In 2022, Naveen isolated the lytic *Salmonella* phage NINP13076 and took it orally for five days, verifying that the oral phage did not damage the probiotic intestinal microbiota (Wang et al., 2022). In the same year, Xinwu investigated a combined strategy using a phage cocktail and the probiotic *Lactobacillus reuteri* to control *S. Typhimurium*. *In vitro* assays showed that the combination effectively eliminated *S. Typhimurium*, indicating a synergistic antibacterial effect. The expression of the inflammatory factors IL-6, IL-1β and TNF-*α* decreases significantly (Kumar et al., 2022). All the above findings prove the positive effects of phages combined with probiotics.

### 5.2 Phage vaccines interactions

Beyond their use as standalone interventions, phages have also been investigated for their compatibility with vaccination strategies, particularly to assess whether phage administration interferes with vaccine-induced immunity. In addition, Kimminau verified through *in vitro* experiments on broiler chickens that dietary phages do not interfere with the colonization or protection of *Salmonella* live vaccines (Kimminau et al., 2022). To determine the adaptive immune response to *S. Enteritidis*, an oral vaccine was administered after exposure to the phage. The results showed that vaccination with a live Enteritidis phage type 4 vaccine could prevent early systemic invasion of *S. typhi* and *S. infantis* (Springer et al., 2021).

### 5.3 Probiotic vaccines synergies

In addition to phage-based combinations, probiotics have also been explored as adjuncts to vaccination strategies to enhance host immune responses and improve protective efficacy against *Salmonella* infection. Graham attempted to use probiotics combined with a recombinant attenuated *Salmonella* live vaccine for verification. The results show that probiotics can improve the bactericidal activity of innate effector cells in whole blood and that the live vaccine RASV can stimulate the bactericidal activity of probiotics, indicating the feasibility of combined therapy (Redweik et al., 2020). Peter J. added a probiotic to the daily drinking water of chickens and injected it intramuscularly at ten weeks of age as a live aro-A deletion mutant vaccine. The vaccine helps limit excretion at sexual maturity and reduces susceptibility to subsequent challenges. The use of probiotics enhances the protective capabilities of vaccines (Groves et al., 2021).

## 6 Broader perspectives and challenges

The successful translation of biological therapies for salmonellosis requires not only scientific innovation but also careful consideration of public health frameworks, regulatory pathways, and commercial feasibility.

The control of Salmonellosis requires a One Health approach that integrates human, animal, and environmental health (Alves et al., 2023). Biological therapies, including bacteriophages, probiotics, and vaccines, can intervene at multiple points in the transmission cycle-reducing pathogen loads in livestock, decontaminating food products, and limiting environmental reservoirs. By decreasing reliance on antibiotics in agriculture, these strategies help mitigate antimicrobial resistance. Additionally, integrated surveillance tools such as whole genome sequencing enable cross-sector monitoring of *Salmonella* strains and resistance determinants, supporting targeted and timely interventions (Liu et al., 2024).

The translation of microbial therapies from research to clinical or veterinary use is heavily influenced by evolving regulatory frameworks. Phage therapy, probiotics, and engineered live biotherapeutics often face complex classification issues, as they do not fit neatly into traditional drug or biologic categories. Regulatory pathways differ across regions, requiring demonstration of safety, efficacy, and manufacturing consistency through well-controlled trials. Engineered phages, in particular, introduce additional biosafety concerns, such as potential effects on commensal microbiota and environmental dissemination, highlighting the need for adaptive oversight and risk mitigation strategies (Fürst-Wilmes et al., 2025; Cordaillat-Simmons et al., 2020).

Beyond regulatory approval, large-scale deployment of microbial therapies faces significant commercialization hurdles. Phage preparations, especially cocktails, must maintain quality, stability, and cost-effectiveness during production and storage. Probiotics and live biotherapeutic products must overcome host-dependent variability and differentiate from general wellness supplements within a still emerging market. High R&D costs, specialized manufacturing requirements, and uncertainties in market adoption and reimbursement increase investment risk, collectively slowing the translation of promising biological interventions into widely accessible therapies (König, 2025).

## 7 Summary, conclusions, and future outlook

The emergence and global spread of multidrug-resistant (MDR) *Salmonella* strains have rendered conventional antibiotic treatments increasingly ineffective, posing a significant threat to public and animal health and incurring substantial economic costs. This escalating crisis has catalyzed an urgent search for alternative therapeutic and preventive strategies. Biological therapies-encompassing bacteriophages, probiotics (including next-generation probiotics and postbiotics), and vaccines-have emerged at the forefront of this endeavor, offering diverse mechanisms of action and considerable promise for combating salmonellosis.

Significant progress has been made across all three modalities. Bacteriophage therapy has advanced from using single, naturally occurring phages to employing sophisticated phage cocktails, microencapsulated formulations for enhanced delivery, and synergistic combinations with antibiotics. The development of engineered phages with tailored properties (e.g., expanded host range using CRISPR-Cas or BRED) and phage-derived lytic enzymes like endolysins represents a major leap toward more potent and predictable phage-based treatments. These approaches not only directly lyse *Salmonella* but can also disrupt biofilms and favorably modulate host immune responses.

Probiotic research has moved beyond general gut health promotion to identify specific strains (*Lactobacillus* spp., *Bifidobacterium* spp., *L. rhamnosus* GG, *L. plantarum*) with well-defined anti-*Salmonella* mechanisms, including competitive exclusion, production of antimicrobial substances (like organic acids and bacteriocins), enhancement of intestinal barrier integrity, and sophisticated immunomodulation (e.g., regulation of cytokine profiles and the NLRP3 inflammasome). The advent of next-generation probiotics, engineered strains designed for specific therapeutic tasks, and particularly postbiotics (the non-viable components and metabolites of probiotics), offers solutions to the stability, viability, and safety concerns associated with live microorganisms, making them highly attractive for broader application.

Vaccine development against *Salmonella* is also undergoing a transformation. While traditional live-attenuated, inactivated, and subunit vaccines have laid the groundwork, novel platforms are gaining traction. These include mRNA vaccines, leveraging technology from viral vaccinology, and nanoparticle-based delivery systems that enhance antigen stability, immunogenicity, and mucosal delivery. Crucially, strategies to achieve broad protection against diverse NTS serovars are advancing through the development of multivalent vaccines, including those based on bacterial ghosts and antigens identified via reverse vaccinology and AI-driven computational methods.

Despite these promising advancements, substantial challenges remain. For all biotherapies, rigorous and large-scale clinical trials are needed to unequivocally establish safety and efficacy, particularly for NTS vaccines and novel phage and probiotic/LBP formulations in human populations. The translation from laboratory promise to widespread clinical and veterinary use is hampered by evolving and often complex regulatory landscapes that are still adapting to the unique nature of these living or biologically derived therapeutics. Commercialization hurdles, including manufacturing scalability and consistency, cost of goods, intellectual property considerations, and market acceptance, also need to be systematically addressed. The inherent biological variability of host–microbe interactions means that personalized or stratified approaches may be necessary for optimal outcomes with some biotherapies.

Future research on *Salmonella* biological therapies should prioritize the development of engineered phages with broader host ranges and resistance to bacterial defenses, optimized cocktail design, targeted delivery, and thorough evaluation of long-term safety, immunogenicity, and ecological impact. For probiotics and derived products, emphasis should be placed on elucidating molecular mechanisms, validating well-characterized strains and postbiotics in diverse host microbiomes, and improving stability and gut-targeted delivery. Vaccine research should focus on broadly protective multivalent formulations, novel platforms such as mRNA and nanoparticles, optimized adjuvants, and large-scale efficacy trials in humans and animals. Combination therapies-including phage-probiotic, phage-vaccine, and probiotic-vaccine approaches-require systematic exploration of synergistic mechanisms and optimal dosing and timing. Cross-cutting priorities include the development of rapid diagnostics to guide therapy, identification of reliable biomarkers, harmonization of regulatory frameworks through international collaboration, and evaluation of cost-effectiveness to support integration into public health and agricultural practices.

The future of salmonellosis biotherapy likely lies in the convergence of multiple advanced technologies, including synthetic biology for engineering microbes and phages, nanotechnology for sophisticated delivery systems, artificial intelligence and machine learning for discovery and prediction, and high-throughput ‘omics technologies to generate system-level insights and mechanistic hypotheses that can be further validated by functional studies. While basic research remains vital, a greater emphasis on translational science-bridging the gap from laboratory discovery to validated clinical application and regulatory approval-is critical for these biotherapies to realize their full potential.

In conclusion, biological therapies represent a vibrant and rapidly evolving frontier in the fight against MDR *Salmonella*. The scientific ingenuity demonstrated in developing engineered phages, targeted probiotics, advanced vaccine platforms, and combination strategies holds immense potential to transform the prevention and treatment of Salmonellosis. Continued multidisciplinary research, robust clinical validation, international collaboration, and strategic investment are essential to overcome the remaining challenges and translate these promising approaches into effective, accessible, and sustainable solutions that can safeguard public and animal health within a comprehensive One Health paradigm.i.
