# Correction: Biological treatments for zoonotic salmonellosis: an evolving therapeutic landscape

**DOI:** 10.3389/fmicb.2026.1856847

**Published:** 2026-05-06

**Authors:** Sushan Ma, Xingmei Liu, Min Shen, Shanshan Deng, Chengxiang Ding, Xin Yang, Lin Zhang, Xu Jia

**Affiliations:** 1 Key Laboratory of Non-coding RNA and Drug Discovery at Chengdu Medical College of Sichuan Province, School of Basic Medical Sciences, Chengdu Medical College, Chengdu, Sichuan, China; 2 Vincent Mary School of Engineering, Science and Technology, Assumption University of Thailand, Bangkok, Thailand; 3 Academy of Animal Husbandry and Veterinary Science, Qinghai University, Xining, Qinghai, China; 4 Department of Pharmacy, ShaoXing People's Hospital, ShaoXing Hospital, ZheJiang University School of Medicine, Shaoxing, Zhejiang, China

**Keywords:** bacteriophages, biotherapies, probiotics, *Salmonella*, salmonellosis, vaccines

The funder [National Natural Science Foundation of China (Grant Number 2026NSFSC1051)] to Xing mei Liu was erroneously omitted.

The corrected **Funding** statement is below:

“The author(s) declared that financial support was received for this work and/or its publication. This work was supported by the Sichuan Province Regional Cooperation Project (Grant No.2023YFQ0075), The National Natural Science Foundation of China (Grant Nos. 32170119, 2026NSFSC1051) to Xingmei Liu, and Funded by CMC Excellent-talent Program (2024kjTzn01).”

Affiliation [Department of Pharmacy, ShaoXing People's Hospital, ShaoXing Hospital, ZheJiang University School of Medicine, Shaoxing, Zhejiang, China] was erroneously given as [Department of Clinical Pharmacy, ShaoXing People's Hospital, ShaoXing Hospital of ZheJiang University, Shaoxing, Zhejiang, China].

The original version of this article has been updated.

